# Association between Maternal Serum Perfluoroalkyl Substances during Pregnancy and Maternal and Cord Thyroid Hormones: Taiwan Maternal and Infant Cohort Study

**DOI:** 10.1289/ehp.1306925

**Published:** 2014-02-21

**Authors:** Yan Wang, Walter J. Rogan, Pau-Chung Chen, Guang-Wen Lien, Hsiao-Yen Chen, Ying-Chih Tseng, Matthew P. Longnecker, Shu-Li Wang

**Affiliations:** 1 Epidemiology Branch, National Institute of Environmental Health Sciences, National Institutes of Health, Department of Health and Human Services, Research Triangle Park, North Carolina, USA; 2 Institute of Occupational Medicine and Industrial Hygiene, and; 3 Department of Public Health, National Taiwan University College of Public Health, Taipei, Taiwan; 4 Department of Environmental and Occupational Medicine, National Taiwan University College of Medicine and National Taiwan University Hospital, Taipei, Taiwan; 5 Division of Environmental Health and Occupational Medicine, National Health Research Institutes, Miaoli, Taiwan; 6 Department of Obstetrics and Gynecology, Hsinchu Cathay General Hospital, Hsinchu, Taiwan; 7 Department of Public Health, College of Public Health, China Medical University, Taichung, Taiwan

## Abstract

Background: Perfluoroalkyl substances (PFASs) are synthetic compounds that are widely used in industry and are often detectable in humans. In pregnant rats and their pups, PFASs can interfere with thyroid hormone homeostasis. In humans, maternal thyroid hormones supply the fetus throughout pregnancy, and thyroid hormones play a critical role in fetal growth and neurodevelopment.

Objectives: We investigated the association between maternal PFAS exposure and thyroid hormone status in pregnant women and neonates.

Methods: In a study of environmental exposure and health in Taiwan, we measured serum concentrations of nine PFASs and four thyroid hormones for 285 pregnant women in their third trimester, and also measured cord serum thyroid hormones for 116 neonates. Associations between maternal PFASs and maternal and cord thyroid hormones were examined in multiple linear regression models.

Results: Perfluorohexanesulfonic acid concentrations were positively associated with maternal thyroid-stimulating hormone (TSH) levels. Pregnant women with higher levels of perfluorononanoic acid (PFNA), perfluoroundecanoic acid (PFUnDA), and perfluorododecanoic acid (PFDoDA) had lower free thyroxine (T_4_) and total T_4_ levels. For example, we estimated that maternal free T_4_ levels decreased 0.019 ng/dL (95% CI: –0.028, –0.009) with each nanogram per milliliter increase in maternal PFNA. Finally, maternal PFNA, PFUnDA, and PFDoDA levels were associated with lower cord total triiodothyronine (T_3_) and total T_4_ levels, and maternal perfluorodecanoic acid (PFDeA) was associated with lower cord total T_3_.

Conclusions: Our results suggest that exposure to some PFASs during pregnancy may interfere with thyroid hormone homeostasis in pregnant women and fetuses.

Citation: Wang Y, Rogan WJ, Chen PC, Lien GW, Chen HY, Tseng YC, Longnecker MP, Wang SL. 2014. Association between maternal serum perfluoroalkyl substances during pregnancy and maternal and cord thyroid hormones: Taiwan Maternal and Infant Cohort Study. Environ Health Perspect 122:529–534; http://dx.doi.org/10.1289/ehp.1306925

## Introduction

Perfluoroalkyl substances (PFASs) are synthetic compounds that are heat resistant and chemically stable. They are widely used in commercial products, such as furniture, carpets, fire-fighting foams, food-packing materials, and cooking utensils (Lau et al. 2007).

Several different pathways have been suggested by which humans are exposed to PFASs, including diet, drinking water, migration from packaged foods, indoor dust, and outdoor air (Fromme et al. 2009). Some PFASs can accumulate in the environment and are often detectable in the tissues of humans (Houde et al. 2006). Long elimination half-lives have been observed for some PFASs in humans: for example, means of 4.8 years for perfluorooctane sulfonate (PFOS) and 3.5 years for perfluorooctanoic acid (PFOA) (Olsen et al. 2007). Although PFOS and PFOA have been phased out by manufacturers, perfluorononanoic acid (PFNA) concentrations have increased in the environment and in the tissues of humans (Andersen et al. 2008; Calafat et al. 2007). The persistence and bioaccumulative properties of PFASs have raised concerns about exposure to PFASs and human health effects.

Experimental studies have found that PFOS treatment reduces total serum concentrations of thyroxine (T_4_) and triiodothyronine (T_3_) in pregnant rats (Chang et al. 2008; Luebker et al. 2005; Thibodeaux et al. 2003), and total T_4_ levels were reduced in rat pups treated with PFOS (Lau et al. 2003; Luebker et al. 2005). For a human study, Kim et al. (2011) recruited pregnant women from hospitals in three large cities in South Korea and analyzed 44 blood samples collected in the third trimester and from the cord at delivery. Their results showed inverse correlations between maternal PFOS and cord T_3_ levels, inverse correlations between maternal perfluorotridecanoic acid (PFTrDA) and cord T_4_ and T_3_, and positive correlations between maternal PFOA and cord thyroid-stimulating hormone (TSH) levels after adjustment (Kim et al. 2011). However, a case–control study of Canadian pregnant women reported that serum concentrations of PFOA, PFOS, and perfluorohexanesulfonic acid (PFHxS) were not associated with hypothyroxinemia (Chan et al. 2011).

Thyroid hormones are essential for normal fetal growth and development. The fetus is completely reliant on maternal T_4_ during the first trimester; thereafter, the fetal thyroid gland begins to function (Contempre et al. 1993; Vulsma et al. 1989). At birth, approximately 30% of T_4_ in cord blood originates from the mother (Fisher 1997). Therefore, both mothers and fetuses supply thyroid hormones during pregnancy. Concerns thus arise about the potential effect of *in utero* PFAS exposure on thyroid hormone homeostasis in pregnant women and their fetuses.

Our study was designed to examine possible associations between maternal serum concentrations of PFASs during pregnancy and *a*) thyroid hormones in pregnant women, and *b*) thyroid hormones in neonates.

## Materials and Methods

*Subjects and data collection*. Our subjects were from a longitudinal birth cohort study of environmental exposures and health among pregnant women and children in central Taiwan, part of the nationwide Taiwan Maternal and Infant Cohort Study. From December 2000 through November 2001, we invited all pregnant women visiting the local medical centers to participate in the study. At their first visit in the obstetric clinic, we recruited 430 women and interviewed them about demographic factors, reproductive and medical histories, and diet during pregnancy. We collected blood samples during their third trimester and umbilical cord blood samples at delivery. Among 430 recruited women, 135 had no measurement of either PFASs or thyroid hormones, and 10 reported thyroid disease; they were excluded from the study. Thus, we had 285 subjects with data on both PFAS concentrations and thyroid hormones.

The Human Ethical Committee of the National Health Research Institutes in Taiwan approved the study. Each of the participants signed informed consent documents at the time of enrollment.

*Exposure assessment*. We sent maternal serum samples to National Taiwan University for measurement of PFASs. Altogether, nine PFASs were analyzed: PFHxS, PFOA, PFOS, PFNA, perfluorodecanoic acid (PFDeA), perfluoroundecanoic acid (PFUnDA), perfluorododecanoic acid (PFDoDA), perfluoroheptanoic acid (PFHpA), and perfluorohexanoic acid (PFHxA). The analytical method has been described elsewhere in detail (Lien et al. 2011; Lin et al. 2013). In this study, we modified the sample preparation procedure slightly. One hundred microliters of plasma sample was vortexed with 100 μL of 1% formic acid (pH = 2.8) for 30 sec. Then 250 μL of methanol and 50 μL of 0.01 ng/mL internal standard solution (13C_8_-PFOA) were added to each sample before the second vortex. The mixture was sonicated for 20 min and then centrifuged at 14,000 rpm for 20 min. The supernatant was collected and then was filtered through a 0.22-μm PVDF syringe filter into a 2.0 mL autosampler vial.

Nine calibration standard solutions were prepared in 100 μL of bovine plasma and were prepared as described. The concentrations of specific analytes ranged from 0.25 to 125 ng/mL, with a fixed amount of internal standard (5 ng/mL).

The separation and detection were performed on an Agilent-1200 high performance liquid chromatography system (Agilent, Palo Alto, CA, USA) coupled with a triple-quadrupole mass spectrometer (Sciex API 4000; Applied Biosystems, Foster City, CA, USA). The limits of quantitation (LOQs), defined as a signal-to-noise ratio of 10, ranged from 0.07 to 0.45 ng/mL for the nine PFASs. The intra-assay coefficients of variation (CVs) for PFAS concentrations ranged from 0.83 to 7.94%, and the interassay CVs were 1.57–24.7%.

*Assessment of thyroid hormones*. Serum concentrations of maternal and cord thyroid hormones, including free T_4_, total T_4_, total T_3_, and TSH, were measured at the central laboratory of Kaohsiung Medical University Hospital using radioimmunoassays. Commercial kits for total T_3_, total T_4_, and TSH were purchased from Daiichi Radioisotope Laboratory (Tokyo, Japan). Free T_4_ kits were from CIS Bio International US Inc. (Bedford, MA, USA). The intraassay CVs of these measures were all < 5% and the interassay CVs were all < 10%.

*Statistical analysis*. The distributions of maternal PFAS concentrations were skewed; therefore, we used medians and 25th, 75th, and 95th percentiles to describe their distributions and a Spearman correlation coefficient (*r*_S_) to describe the pair-wise relationships among maternal PFASs. Maternal and cord T_4_ concentrations were normally distributed. Maternal and cord total T_3_ became normally distributed after removal of a single unusually high value from each distribution. Maternal and cord free T_4_ and TSH were skewed. Thus we used means ± SD and medians [inter-quartile range (IQR)] to describe their distributions. We compared means between two groups with Student’s *t*-test and compared frequencies between groups with a chi-square test. For PFAS values below the LOQ, we imputed the corresponding expected value (E) conditional on values being below LOQ based on an assumed log-normal distribution of PFASs (Richardson and Ciampi 2003) and used these when fitting models.

In preliminary analyses (not shown), we used a generalized additive model with a cubic smoothing spline to evaluate the possibility of a nonmonotonic relationship between each maternal PFAS (untransformed) and each outcome; a significant spline term for a given maternal PFAS would indicate a nonlinear relationship with the outcome. We did not find any significant spline terms (*p* > 0.05) in any model; therefore, we fit a linear regression model to each maternal thyroid hormone in relation to each maternal PFAS concentration and for each cord thyroid hormone and each maternal PFAS. We made no general adjustment for multiple comparisons (Rothman 1990).

In the linear regression models, thyroid hormones were the dependent variables and maternal PFASs were continuous independent variables. Thus the linear coefficient (β) corresponds to the unit change in the thyroid hormone with a 1-unit (nanograms per milliliter) increase in maternal PFAS. We present results with and without natural log transformation (ln-transformation) of the thyroid hormones. Additionally, we express the estimated effect size as the ratio (percent) of β to the mean thyroid hormone concentration, which can be interpreted as an index of association size.

Covariates considered were maternal age, maternal education, previous live births, family income, maternal prepregnancy body mass index (BMI; kilograms per meter squared), and maternal fish consumption during pregnancy. We included maternal age at enrollment (continuous) *a priori* in the models, and then we used univariate regression to identify covariates that predicted both maternal PFAS concentrations and maternal thyroid hormone concentrations with *p* < 0.1. Factors included in the final model based on this criterion were maternal education (< high school, high school, part or full college, and > college) and previous live births (0 and ≥ 1). When we fit models between maternal PFASs and cord thyroid hormones, we also adjusted for the neonate’s sex and type of delivery (normal vaginal, vacuum, and cesarean section). Maternal fish consumption might be associated with both maternal PFASs and maternal thyroid hormones; however, adjustment for it may result in overadjustment because fish are also a source of PFASs. Fish are also a source of other contaminants, such as polychlorinated biphenyls (PCBs), and iodine, both of which can affect thyroid hormone levels. Therefore, we performed sensitivity analyses by comparing results with and without adjustment for maternal fish consumption during pregnancy.

We searched for influential observations in each of the linear regression models (Belsley et al. 1980). There were few influential observations (*n* < 10) in any model and no major differences were found in the results after they were excluded. Therefore, we present only the results with all data included. We used SAS (version 9.2; SAS Institute Inc., Cary, NC, USA) to perform all statistical analysis. We considered *p*-values < 0.05 to indicate statistical significance.

## Results

*Participant characteristics*. The characteristics of the 285 pregnant women are shown in Table 1. They averaged 29 years of age, had an average prepregnancy BMI of 20.6 kg/m^2^, and generally did not smoke or consume alcohol during pregnancy. Almost all had high school education or more, and more than half of the women were primiparous. The 285 subjects, compared with the 135 who had no data on PFAS and thyroid hormones, were not significantly different in age, prepregnancy BMI, fish consumption, previous live births, or education.

**Table 1:** Characteristics of the pregnant women (*n* = 285) and neonates (*n* = 116).

Characteristic	Mean ± SD or *n* (%)
Maternal age at enrollment (years)	28.8 ± 4.3
Maternal weight (kg)	52.3 ± 7.7
Maternal height (m)	159.2 ± 4.7
Maternal prepregnancy BMI (kg/m^2^)	20.6 ± 2.9
Maternal fish consumption (times/week) [median (IQR)]	3.8 (1.9, 6.8)
Maternal previous live births	
0	162 (57.0)
≥ 1	123 (43.0)
Maternal smoking during pregnancy	4 (1.4)
Maternal drinking alcohol during pregnancy	2 (0.7)
Maternal education	
< High school	16 (5.6)
High school	127 (44.4)
Part or full college	100 (35.2)
> College	42(14.8)
Maternal annual family income (US$)	
< 13, 771	42 (15.1)
≥ 13, 771 to < 20, 672	84 (30.1)
≥ 20, 672 to < 34, 453	111 (39.4)
≥ 34, 453	43 (15.4)
Neonatal sex	
Female	59 (50.9)
Male	57 (49.1)
Neonatal type of delivery	
Normal vaginal	40 (34.5)
Vacuum	42 (36.2)
Cesarean section	34 (29.3)
Neonatal gestational weeks at birth	38.8 ± 1.6
Neonatal birth weight (kg)	3.2 ± 0.4

Among the singleton infants delivered by these women, 116 had at least one cord serum thyroid hormone measurement (Table 1). Compared with those whose cord measurements were not available (*n* = 314), the distribution by sex and by type of delivery was not significantly different, nor were mean gestational weeks at birth, birth weight or maternal thyroid hormone levels (*n* = 169, ln-transformed). However, neonates with cord measurements had significantly higher mean maternal concentrations of PFNA (2.5 vs. 1.8 ng/mL), PFUnDA (8.7 vs. 5.1 ng/mL), and PFDoDA (0.44 vs. 0.35 ng/mL) (ln-transformed).

*Maternal PFAS and maternal and cord thyroid hormone levels*. Among the nine maternal PFASs, PFHxA and PFHpA concentrations were detected in < 20% of samples and thus were not considered further. The other seven substances were detected in > 70% of the serum samples (Table 2). PFOS had the highest median concentration, followed by PFUnDA, PFOA, PFNA, PFHxS, PFDeA, and PFDoDA. Concentrations of PFNA, PFUnDA, and PFDoDA were highly correlated (*r*_S_ ≥ 0.87, all *p* < 0.001). PFDeA concentrations were moderately correlated with concentrations of PFNA (*r*_S_ = 0.62, *p* < 0.001), PFUnDA (*r*_S_ = 0.57, *p* < 0.001), and PFDoDA (*r*_S_ = 0.58, *p* < 0.001) (see Supplemental Material, Table S1). The serum concentrations of thyroid hormones in the pregnant women and neonates are shown in Table 3. Maternal and cord total T_4_ and total T_3_ were normally distributed. The means and medians of maternal free T_4_, as well as cord free T_4_, were quite close. However, the distributions of both free T_4_ and TSH were skewed. As expected, cord TSH level was high, which is attributed to labor and delivery.

**Table 2:** Serum concentrations of maternal PFASs (ng/mL).

Maternal PFASs (*n*= 285)	Molecular formula	Percent > LOQ	25thpercentile	Median	75thpercentile	95th percentile
Perfluorohexanesulfonic acid (PFHxS)	C_5_F_13_CSO_3_H	78	0.30	0.81	1.35	2.90
Perfluorooctanoic acid (PFOA)	C_7_F_15_CO_2_H	87	1.54	2.39	3.40	5.20
Perfluorooctane sulfonate (PFOS)	C_7_F_17_CSO_3_H	100	9.65	12.73	17.48	27.85
Perfluorononanoic acid (PFNA)	C_8_F_17_CO_2_H	96	0.85	1.51	2.51	6.20
Perfluorodecanoic acid (PFDeA)	C_9_F_19_CO_2_H	71	0.10	0.46	0.69	1.09
Perfluoroundecanoic acid (PFUnDA)	C_10_F_21_CO_2_H	91	1.70	3.26	9.20	22.05
Perfluorododecanoic acid (PFDoDA)	C_11_F_23_CO_2_H	82	0.23	0.36	0.53	0.85

**Table 3:** Serum concentrations of maternal and cord thyroid hormones.

Sample	Free T_4_ (ng/dL)	Total T_4_ (μg/dL)	Total T_3_ (μg/dL)	TSH (μIU/mL)
Pregnant women
*n*	285	274	276	283
Mean ± SD	0.60 ± 0.2	11.29 ± 2.3	0.16 ± 0.04	2.03 ± 1.3
Median (IQR)	0.57 (0.49, 0.69)	11.16 (9.70, 12.80)	0.16 (0.13, 0.18)	1.76 (1.19, 2.45)
Cord
*n*	92	116	112	114
Mean ± SD	0.74 ± 0.2	8.36 ± 2.0	0.06 ± 0.02	9.00 ± 6.9
Median (IQR)	0.73 (0.60, 0.88)	8.37 (6.80, 9.80)	0.05 (0.046, 0.066)	6.65 (4.60, 11.00)

*Association between maternal PFASs and maternal thyroid hormones*. The models showed similar results before and after adjustment for covariates; therefore, the coefficients (βs) and 95% CIs are shown only after adjustment in Table 4. Most maternal PFASs were inversely associated with maternal free T_4_, and the associations with maternal PFNA, PFUnDA, and PFDoDA were statistically significant. According to model estimates, maternal free T_4_ levels decreased 0.019 ng/dL (95% CI: –0.028, –0.009) with each 1-ng/mL increase in maternal PFNA, 0.004 ng/dL (95% CI: –0.007, –0.002) with maternal PFUnDA, and 0.132 ng/dL (95% CI: –0.204, –0.059) with maternal PFDoDA. The index of association size for free T_4_ per unit change in maternal PFASs is given by β/mean free T_4_ × 100: –3.2% for maternal PFNA, –0.7% for PFNA, and –22% for PFUnDA, respectively.

**Table 4:** Linear regression coefficients (95% CI) for associations between maternal PFASs (ng/mL) and maternal thyroid hormones (μIU/mL).^*a*^

Maternal PFAS^*b*^	Free T_4_ (*n *= 285) (ng/dL)	Total T_4_ (*n *= 274) (μg/dL)	Total T_3_ (*n *= 276) (ng/dL)	TSH (*n *= 283)
PFHxS	–0.010 (–0.023, 0.003)	–0.130 (–0.316, 0.057)	–0.002 (–0.005, 0.001)	0.105 (0.002, 0.207)*
PFOA	–0.003 (–0.012, 0.005)	0.011 (–0.108, 0.130)	–0.000 (–0.002, 0.009)	0.011 (–0.057, 0.078)
PFOS	0.001 (–0.002, 0.003)	0.019 (–0.016, 0.053)	0.000 (–0.002, 0.001)	–0.005 (–0.024, 0.013)
PFNA	–0.019 (–0.028, –0.009)^#^	–0.189 (–0.333, –0.046)**	–0.001 (–0.003, 0.002)	0.033 (–0.046, 0.112)
PFDeA	–0.001 (–0.006, 0.005)	0.047 (–0.028, 0.123)	0.002 (0.000, 0.003)**	0.004 (–0.037, 0.045)
PFUnDA	–0.004 (–0.007, –0.002)^#^	–0.062 (–0.097, –0.026)^#^	–0.000 (–0.001, 0.000)	0.011 (–0.009, 0.030)
PFDoDA	–0.132 (–0.204, –0.059)^#^	–1.742 (–2.785, –0.700)**	–0.005 (–0.022, 0.011)	0.365 (–0.215, 0.944)
^***a***^Adjusted for maternal age, maternal education levels, and maternal previous live births. ^***b***^Values below LOQ were imputed based on expected values assuming a log-normal distribution. *p < 0.05. **p < 0.01. ^#^*p* < 0.001.				

Maternal PFNA, PFUnDA, and PFDoDA were also significantly inversely associated with total T_4_. With each 1-ng/mL increase in maternal PFNA, PFUnDA, and PFDoDA, there was an estimated decrease of 0.189 μg/dL (95% CI: –0.333, –0.046), 0.062 μg/dL (95% CI: –0.097, –0.026), and 1.742 μg/dL (95% CI: –2.785, –0.700) in maternal total T_4_, respectively. The index of association size was –1.7%, –0.5%, and –15.4% respectively.

We found a significant positive association between maternal PFDeA and maternal total T_3_, although the magnitude was small (β = 0.002; 95% CI: 0.000, 0.003). Associations between other maternal PFASs and maternal total T_3_ were very small and not statistically significant. Maternal PFHxS was positively associated with maternal TSH levels with statistical significance. There was an estimated increase of 0.105 μIU/mL (95% CI: 0.002, 0.207) in maternal TSH levels with each 1-ng/mL increase in maternal PFHxS. The index of association size was a 5.2% increase in maternal TSH per 1-ng/mL increase in maternal PFHxS.

Results for ln-transformed maternal thyroid hormones are presented in the Supplemental Material, Table S2. There were no differences in the direction or significance of the observed associations between the analyses using transformed or untransformed thyroid hormone values. In the sensitivity analysis, adjustment for maternal fish consumption did not change the results for either maternal or cord thyroid hormones (data not shown).

*Association between maternal PFASs and cord thyroid hormones*. We also investigated the associations between maternal PFASs and cord thyroid hormones (Table 5). Maternal PFNA, PFUnDA, and PFDoDA levels were also inversely associated with cord total T_4_. The cord total T_4_ level decreased 0.213 ng/dL (95% CI: –0.384, –0.042) with each 1-ng/mL increase in maternal PFNA, 0.052 ng/dL (95% CI: –0.095, –0.010) with each 1-ng/mL increase in PFUnDA, and 1.92 ng/dL (95% CI: –3.345, –0.495) with each 1-ng/mL increase in PFDoDA. The index of association size was –2.5%, –0.6%, and –23% with each 1-ng/mL increase in maternal PFNA, PFDoDA, and PFUnDA, respectively.

**Table 5:** Linear regression coefficients (95% CI) for associations between maternal PFASs (ng/mL) and cord thyroid hormones (μIU/mL).^*a*^

Maternal PFAS^*b*^	Free T_4_ (*n *= 92) (ng/dL)	Total T_4_ (*n *= 116) (μg/dL)	Total T_3_ (*n *= 112) (ng/dL)	TSH (*n *= 114)
PFHxS	–0.030 (–0.098, 0.039)	0.002 (–0.495, 0.500)	–0.001 (–0.007, 0.004)	0.493 (–1.449, 2.434)
PFOA	–0.029 (–0.062, 0.004)	0.128 (–0.094, 0.350)	–0.001 (–0.004, 0.001)	–0.498 (–1.464, 0.468)
PFOS	0.001 (–0.006, 0.008)	0.032 (–0.024, 0.087)	0.000 (–0.000, 0.001)	–0.083 (–0.292, 0.127)
PFNA	0.001 (–0.021, 0.023)	–0.213 (–0.384, –0.042)*	–0.002 (–0.004, –0.001)**	–0.361 (–0.955, 0.234)
PFDeA	0.020 (–0.124, 0.164)	–0.513 (–1.732, 0.706)	–0.017 (–0.028, –0.005)**	–3.505 (–7.821, 0.812)
PFUnDA	0.002 (–0.004, 0.007)	–0.052 (–0.095, –0.010)*	–0.001 (–0.001, –0.0002)**	–0.083 (–0.232, 0.066)
PFDoDA	–0.009 (–0.183, 0.165)	–1.920 (–3.345, –0.495)**	–0.022 (–0.035, –0.009)**	–1.539 (–6.582, 3.503)
^***a***^Adjusted for maternal age, maternal education levels, maternal previous children, neonatal sex, and neonatal type of delivery. ^***b***^Values below LOQ were imputed based on expected values assuming a log-normal distribution. *p < 0.05. **p < 0.01.				

Additionally, these three substances were also inversely associated with cord total T_3_ levels. With each ng/mL increase in maternal PFNA, PFUnDA, and PFDoDA, there was a decrease of 0.002 μg/dL (95% CI: –0.004, –0.001), 0.001 μg/dL (95% CI: –0.001, –0.0002), and 0.022 μg/dL (95% CI: –0.035, –0.009) in cord total T_3_, respectively. The index of association size was –3.3%, –1.7%, and –0.3% respectively. Higher maternal PFDeA concentrations were also significantly associated with lower cord total T_3_. One 1-ng/mL increase in PFDeA was associated with a 0.017-ng/dL decrease (95% CI: –0.028, –0.005) decrease in cord total T_3_. The index of association size was 24%.

Associations between maternal PFNA and cord total T_4_, and maternal PFUnDA and cord total T_4_, were still negative but lost statistical significance after further adjustment for fish consumption (data not shown), whereas other associations were similar to those without adjustment for fish consumption. Results for ln-transformed cord thyroid hormones were similar (see Supplemental Material, Table S3).

## Discussion

In these data, maternal serum concentrations of three PFASs—PFNA, PFUnDA, and PFDoDA—were strongly correlated. All three were significantly inversely associated with maternal free T_4_ and total T_4_ and with cord total T_3_ and total T_4_. Additionally, we found a statistically significant positive association between maternal serum concentrations of PFHxS and maternal TSH levels, and a significant positive association of maternal PFDeA with maternal total T_3_, but inverse association between maternal PFDeA and cord total T_3_ levels.

The inverse associations we observed between maternal PFNA, PFUnDA, and PFDoDA and maternal free T_4_ and total T_4_ and cord total T_3_ and total T_4_ have not been reported before. A study done in South Korea, where pregnant women were recruited from three hospitals in large cities and their blood samples (*n* = 44) were collected in the third trimester and from the cord at delivery, reported inverse associations between maternal PFOS and cord T_3_ levels, and between maternal PFTrDA and cord T_4_ and T_3_ (Kim et al. 2011). However, we did not find a significant adverse association between maternal PFOS and cord T_3_. Because we did not measure maternal PFTrDA, we could not replicate that finding. In nonpregnant women, some PFASs were found to be associated with elevated T_4_ (Dallaire et al. 2009; Knox et al. 2011).

We found a significant positive association between maternal PFDeA and maternal total T_3_, but it was small (β = 0.002). The association between maternal PFDeA and cord total T_3_ was negative, which is consistent with the inverse associations between maternal PFNA, PFUnDA, and PFDoDA and thyroid hormones in both maternal and cord serum. Thus, we believe the positive association is likely attributable to chance.

A reduction in serum T_4_ level can cause a feedback elevation of TSH level. We observed positive associations between maternal PFNA, PFUnDA, and PFDoDA and maternal TSH, although nonsignificant. However, there was a positive association between maternal PFHxS and maternal TSH, and adverse, though nonsignificant, associations between maternal PFHxS and maternal free T_4_ and total T_4_. In the study of Korean women conducted by Kim et al. (2011), there was a positive correlation between maternal PFOA and cord TSH levels. In the two available rodent studies, no significant changes in serum TSH levels were detected among pregnant rats treated with PFOS (Lau et al. 2003) or among PFOS-exposed pups (Thibodeaux et al. 2003) despite decreases in total T_4_ in both studies. However, Luebker et al. (2005) reported higher serum TSH in association with lower T_4_ among rat pups from PFOS-treated dams, and Seacat et al. (2002) also found an increase in serum TSH among both male and nonpregnant female monkeys with treatment of PFOS.

To our knowledge, there are no studies in experimental animals on the mechanism by which PFNA, PFUnDA, and PFDoDA might modify thyroid hormones. However, investigations have been carried out for PFOS in rats. PFOS caused a reduction in both total T_4_ and free T_4_ levels in pregnant rats, in a protocol with daily dosing from gestational day 2 to day 20 (Thibodeaux et al. 2003). In another study, a single dose of 15 mg of PFOS (producing a concentration in serum of approximately 50–75 μg/mL) lowered total T_3_ and total T_4_ (Chang et al. 2008). It was hypothesized that PFOS competed with free T_4_ for binding sites on the thyroid hormone transport protein transthyretin (TTR) in rats, which may have resulted in a lowering of serum total T_3_ and total T_4_ (Chang et al. 2008). Such competitive displacement may also occur in humans, a suggestion supported by an *in vitro* study in which PFASs exhibited binding potency with human TTR (Weiss et al. 2009). However, different PFASs showed different binding. PFNA, PFUnDA, and PFDoDA showed less binding strength than PFOS and PFOA (Weiss et al. 2009). This indicates that protein binding is probably not the sole factor contributing to the variation in associations of PFASs with thyroid hormones.

Besides affecting thyroid hormone transport, PFASs may also alter thyroid hormones biosynthesis and metabolism. In a study by Yu et al. (2009), hepatic uridine diphosphoglucuronosyl transferase 1A1 (UGT1A1) activity, which is involved in hepatic metabolism of thyroid hormones, was induced in PFOS-exposed rats and subsequently led to glucuronidation of T_4_ and circulating T_4_ deficiency. Apart from UGT1A1, the authors also found that PFOS increased thyroidal conversion of T_4_ to T_3_ via type 1 deiodinase, which was also a contribution to the reduction in T_4_ (Yu et al. 2009). The PFAS concentrations in treated rats were much higher than seen in the general population of humans.

The median serum concentrations of long-chain PFASs, PFNA, PFUnDA, and PFDoDA were slightly higher in our Taiwan study population than reported for study populations in western countries (Calafat et al. 2007; Gutzkow et al. 2012), but comparable with levels found in populations from Japan, Korea, and Vietnam (Harada et al. 2011). However, two other studies from Taiwan reported much higher concentrations than we observed (Lien et al. 2011; Lin et al. 2013), suggesting that local or regional sources of exposure may be important in Taiwan. Additionally, concentrations of maternal PFNA, PFUnDA, and PFDoDA were strongly correlated, indicating that these substances may have common sources of exposure. Because these are longer-chain PFASs, which are considered more bioaccumulative and toxic than shorter-chain PFASs (Goecke-Flora and Reo 1996; Kudo et al. 2001), investigation is needed on their sources, routes of exposures, and any resulting toxicity.

We had only one maternal serum sample and one cord serum sample (representing the infant), with which to assess both exposure and outcome. This leads to the question of whether these samples allow any reasonable inferences. In another population of Danish pregnant women, concentrations of PFOS and PFOA in samples drawn in the first and second trimester are highly correlated (both 0.9) (Fei et al. 2007). Concentrations do decline over the course of pregnancy, but our samples were all drawn in a single (the third) trimester. If the other PFASs behave like PFOS and PFOA, it seems reasonable to regard women with higher concentrations in the samples we have as being more highly exposed. For the maternal outcomes, total T_3_ and total T_4_ are higher during pregnancy and increase over its course; free T_4_ is lower and decreases somewhat; the range of TSH is about the same for pregnant and nonpregnant women (Soldin et al. 2004). Variability in a given woman over the course of pregnancy is reported to be smaller than the variability between women (Boas et al. 2009), so the concentration in a given sample can be viewed as more representative of the individual from whom it was drawn than of the trimester in which it was drawn. Thus, although samples drawn at multiple times might be better, using the sample at a given time during pregnancy does not appear likely to produce spurious associations; it would likely decrease power and attenuate measures of association.

T_3_ and T_4_ increase over gestation, but free T_4_ is stable. Cord blood at term can have the highest T_4_ experienced by the fetus, because TSH normally falls after birth. There is generally not good correlation between T_4_, T_3_, or TSH in cord serum and maternal serum at term; this is thought to result from the increasing autonomy of the fetal thyroid axis as the pregnancy proceeds (Hume et al. 2004). Thus, any biological basis for the associations we see with thyroid hormones in cord serum and maternal PFASs should reflect effects on the fetus, rather than on the mother. Measurement of infant thyroid hormones shortly after birth might have been more informative, because they are less affected by perturbations associated with delivery.

The main strength of the present study was having data for both a panel of PFASs and thyroid hormones. Yet some limitations should be noted. First, although our study was relatively large, the size was not sufficient to evaluate clinical outcomes such as hypothyroidism or hypothyroxinemia. Second, three of the substances, PFNA, PFUnDA, and PFDoDA, were highly correlated, so we were unable to distinguish their independent associations. Third, many factors can affect thyroid hormone levels, such as iodine status or thyroid antibodies, and we did not have measurements of all of them. Finally, some aspects of this study were cross-sectional, making the direction of causation uncertain.

In conclusion, thyroid hormones are of critical importance to both pregnant women and their offspring. Decreased maternal provision of T_4_ to the fetus leads to an increased risk of poor cognition, behavior and growth (Idris et al. 2005; Sahu et al. 2010). PFASs have been reported to be associated with low birth weight (Maisonet et al. 2012; Washino et al. 2009) and developmental problems in childhood (Hoffman et al. 2010), which might be attributable in part to disruption of thyroid hormone homeostasis. Our findings suggest maternal PFAS exposure may interfere with both maternal and fetal thyroid hormone homeostasis. The ubiquity of PFASs and the critical role of thyroid hormones in fetal growth and neurodevelopment make the findings of potentially great public health importance.

## Supplemental Material

(181 KB) PDF
