# Supplementary material for: Association between Maternal Serum Perfluoroalkyl Substances during Pregnancy and Maternal and Cord Thyroid Hormones: Taiwan Maternal and Infant Cohort Study
Source: Environ Health Perspect. 2014 Feb 21;122(5):529–34. doi: 10.1289/ehp.1306925 (PMC4014761; doi:10.1289/ehp.1306925)
Supplement: (181 KB) PDF [file ehp.1306925.s001.pdf]

Supplemental Material

Association between Maternal Serum Perfluoroalkyl Substances during Pregnancy and Maternal and Cord Thyroid Hormones: Taiwan Maternal and Infant Cohort Study

Yan Wang, Walter J. Rogan, Pau-Chung Chen, Guang-Wen Lien, Hsiao-Yen Chen, Ying-Chih Tseng, Matthew P. Longnecker, and Shu-Li Wang

Table of Contents **Page**

Table S1. Spearman Correlation Coefficients among Maternal PFASs. 2

Table S2. Linear Regression Coefficients for Associations between Maternal PFASs and Maternal Thyroid Hormones (ln-transformed). 3

Table S3. Linear Regression Coefficients for Associations between Maternal PFASs and Cord Thyroid Hormones (ln-transformed). 4

Table S1. Spearman Correlation Coefficients among Maternal PFASs.

	**PFHxS**	**PFOA**	**PFOS**	**PFNA**	**PFDeA**	**PFUnDA**	**PFDoDA**
PFHxS	1	0.19*	0.18*	0.11	0.07	-0.01	0.05
PFOA		1	0.43**	0.28**	0.28**	0.17*	0.22*
PFOS			1	0.22*	0.39**	0.11	0.16*
PFNA				1	0.62**	0.89**	0.90**
PFDeA					1	0.57**	0.58**
PFUnDA						1	0.87**
PFDoDA							1

Abbreviations: PFHxS, perfluorohexanesulfonic acid; PFOA, perfluorooctanoic acid; PFOS, perfluorooctane sulfonate; PFNA, perfluorononanoic acid; PFDeA, perfluorodecanoic acid; PFUnDA, perfluoroundecanoic acid; PFDoDA, perfluorododecanoic acid. Units of PFASs are in ng/mL.

* $p < 0.05$, ** $p < 0.001$.

Table S2. Linear Regression Coefficients (95% CI) for Associations between Maternal PFASs and Maternal Thyroid Hormones (ln-transformed).

**Maternal PFAS** [a]	**Free T4 (N = 285)** [b]	**Total T4 (N = 274)** [b]	**Total T3 (N = 276)** [b]	**TSH (N = 283)** [b]
PFHxS	-0.015 (-0.036, 0.007)	-0.010 (-0.027, 0.007)	-0.011 (-0.031, 0.010)	0.061 (0.010, 0.111)*
PFOA	-0.006 (-0.020, 0.008)	0.004 (-0.008, 0.015)	0.001 (-0.013, 0.014)	0.024 (-0.008, 0.056)
PFOS	0.001 (-0.003, 0.005)	0.002 (-0.001, 0.005)	0.003 (-0.001, 0.007)	0.001 (-0.008, 0.010)
PFNA	-0.032 (-0.048, -0.016)***	-0.017 (-0.030,-0.003)*	-0.002 (-0.018, 0.014)	0.026 (-0.013, 0.065)
PFDeA	-0.000 (-0.009, 0.008)	0.004 (-0.003, 0.011)	0.008 (0.000, 0.017)**	0.005 (-0.016, 0.025)
PFUnDA	-0.007 (-0.011, -0.003)***	-0.006 (-0.009,-0.002)***	-0.002 (-0.006, 0.002)	0.005 (-0.004, 0.015)
PFDoDA	-0.217 (-0.335, -0.099)***	-0.149 (-0.246,-0.053)**	-0.030 (-0.146, 0.086)	0.194 (-0.094, 0.482)

Abbreviations: CI, confidence interval; PFHxS, perfluorohexanesulfonic acid; PFOA, perfluorooctanoic acid; PFOS, perfluorooctane sulfonate; PFNA, perfluorononanoic acid; PFDeA, perfluorodecanoic acid; PFUnDA, perfluoroundecanoic acid; PFDoDA, perfluorododecanoic acid; T3, triiodothyronine; T4, thyroxin; TSH, thyroid stimulating hormone. Units of PFASs are ng/mL. Unit of TSH is µIU/mL, units of free T4 and total T3 are ng/dL and unit of total T4 is µg/dL.

[a]Values below LOQ were imputed based on expected values assuming a log-normal distribution. [b]Maternal thyroid hormones were ln-transformed. Numbers varied because of missing data for thyroid hormones. Models were adjusted for maternal age, maternal education levels and maternal previous live births.

*$p < 0.05$; ** $p < 0.01$; *** $p < 0.001$.

Table S3. Linear Regression Coefficients (95% CI) for Associations between Maternal PFASs and Cord Thyroid Hormones (ln-transformed).

**Maternal PFAS** [a]	**Free T4 (N = 92)** [b]	**Total T4 (N = 116)** [b]	**Total T3 (N = 112)** [b]	**TSH (N = 114)** [b]
PFHxS	-0.010 (-0.097, 0.077)	-0.029 (-0.100, 0.042)	-0.007 (-0.099, 0.084)	0.095 (-0.062, 0.252)
PFOA	-0.005 (-0.042, 0.033)	-0.013 (-0.048, 0.023)	-0.020 (-0.066, 0.026)	0.019 (-0.051, 0.089)
PFOS	0.004 (-0.006, 0.013)	0.002 (-0.006, 0.009)	0.003 (-0.007, 0.012)	0.001 (-0.016, 0.019)
PFNA	0.002 (-0.030, 0.034)	-0.022 (-0.044, -0.000)*	-0.041 (-0.068, -0.014)**	-0.014 (-0.068, 0.040)
PFDeA	0.065 (-0.136, 0.266)	-0.125 (-0.284, 0.035)	-0.325 (-0.522, -0.127)**	-0.183 (-0.560, 0.193)
PFUnDA	0.002 (-0.006, 0.010)	-0.006 (-0.011, -0.000)*	-0.012 (-0.018, -0.005)***	-0.004 (-0.017, 0.010)
PFDoDA	0.022 (-0.230, 0.273)	-0.223 (-0.404, -0.042)*	-0.408 (-0.636, -0.181)***	0.026 (-0.430, 0.482)

Abbreviations: CI, confidence interval; PFHxS, perfluorohexanesulfonic acid; PFOA, perfluorooctanoic acid; PFOS, perfluorooctane sulfonate; PFNA, perfluorononanoic acid; PFDeA, perfluorodecanoic acid; PFUnDA, perfluoroundecanoic acid; PFDoDA, perfluorododecanoic acid; T3, triiodothyronine; T4, thyroxin; TSH, thyroid stimulating hormone. Units of PFASs are ng/mL. Unit of TSH is µIU/mL, units of free T4 and total T3 are ng/dL and unit of total T4 is µg/dL.

[a]Values below LOQ were imputed based on expected values assuming a log-normal distribution. [b]Maternal thyroid hormones were ln-transformed. Numbers varied because of missing data with thyroid hormones. Models were adjusted for maternal age, maternal education levels and maternal previous live births.

* $p < 0.05$; ** $p < 0.01$; *** $p <0.001$.
